# Postoperative adjuvant radiochemotherapy with cisplatin versus adjuvant radiochemotherapy with cisplatin and pembrolizumab in locally advanced head and neck squamous cell carcinoma- the study protocol of the Adrisk trial

**DOI:** 10.3389/fonc.2023.1128176

**Published:** 2023-03-21

**Authors:** Susanne Wiegand, Gunnar Wichmann, Jeannette Vogt, Kathrin Vogel, Annegret Franke, Thomas Kuhnt, Florian Lordick, Anne-Marie Scheuble, Peter Hambsch, Peter Brossart, Franz Georg Bauernfeind, Holger Kaftan, Georg Maschmeyer, Matthias Paland, Marc Münter, Victor Lewitzki, Nicole Rotter, Carmen Stromberger, Marcus Beck, Steffen Dommerich, Thomas Christoph Gauler, Gunnar Hapke, Orlando Guntinas-Lichius, Ursula Schröder, Martin Görner, Matthias G. Hautmann, Felix Steger, Bálint Tamaskovics, Anett Schmiedeknecht, Andreas Dietz

**Affiliations:** ^1^ Department of Otolaryngology, Head and Neck Surgery, Leipzig University Medical Center, Leipzig, Germany; ^2^ Clinical Trial Centre Leipzig, University of Leipzig, Leipzig, Germany; ^3^ Department of Radiation Oncology, University Hospital Leipzig, Leipzig, Germany; ^4^ University Cancer Center Leipzig, Leipzig University Medical Center, Leipzig, Germany; ^5^ Department of Oncology, Hematology, Immuno-Oncology, Rheumatology and Clinical Immunology, University Hospital Bonn, Bonn, Germany; ^6^ Department of Otorhinolaryngology, Helios-Klinikum Erfurt, Erfurt, Germany; ^7^ Department of Haematology, Oncology, and Palliative Care, Ernst Von Bergmann Clinic, Potsdam, Germany; ^8^ Institute of Radiotherapy, Klinikum Stuttgart, Stuttgart, Germany; ^9^ Department of Radiation Oncology, University of Würzburg, Würzburg, Germany; ^10^ Department of Otorhinolaryngology, Head and Neck Surgery, University Medical Center Mannheim, Mannheim, Germany; ^11^ Department of Radiation Oncology, Charité, Berlin, Germany; ^12^ Vivantes Klinikum Neukölln, Department of Radiooncology and Radiotherapy, Berlin, Germany; ^13^ Department of Otolaryngology, Charité, Berlin, Germany; ^14^ Department of Radiotherapy, West German Cancer Center, University Hospital Essen, University Duisburg-Essen, Essen, Germany; ^15^ Department of Hematology and Oncology, Marienkrankenhaus Hamburg, Hamburg, Germany; ^16^ Department of Otorhinolaryngology, Jena University Hospital, Jena, Germany; ^17^ Department of Otorhinolaryngology, University of Lübeck, Lübeck, Germany; ^18^ Department of Hematology, Oncology and Palliative Medicine, Klinikum Bielefeld Mitte, Bielefeld, Germany; ^19^ Department for Radiotherapy, University Hospital Regensburg, Regensburg, Germany; ^20^ Department of Radiation Oncology, Medical Faculty and University Hospital Düsseldorf, Heinrich Heine University, Düsseldorf, Germany

**Keywords:** head neck cancer, immunotherapy, pembrolizumab, immune checkpoint blockade, PD-1:PD-L1 axis, upfront surgery, locoregional disease, randomized controlled phase IIB clinical trial

## Abstract

Most of the patients with head and neck squamous cell carcinoma (HNSCC) are diagnosed with locally advanced disease. Standards of care for curative-intent treatment of this patient group are either surgery and adjuvant radio(chemo)therapy (aRCT) or definitive chemoradiation. Despite these treatments, especially pathologically intermediate and high-risk HNSCC often recur. The ADRISK trial investigates in locally advanced HNSCC and intermediate and high risk after up-front surgery if the addition of pembrolizumab to aRCT with cisplatin improves event-free sur-vival compared to aRCT alone. ADRISK is a prospective, randomized controlled investiga-tor-initiated (IIT)-phase II multicenter trial within the German Interdisciplinary Study Group of German Cancer Society (IAG-KHT). Patients with primary resectable stage III and IV HNSCC of the oral cavity, oropharynx, hypopharynx and larynx with pathologic high (R1, extracapsular nodal extension) or intermediate risk (R0 <5 mm; N≥2) after surgery will be eligible. Two hun-dred forty patients will be randomly assigned (1:1) to either standard aRCT with cisplatin (standard arm) or aRCT with cisplatin + pembrolizumab (200 mg iv, in 3-week cycle, max. 12 months) (interventional arm). Endpoints are event-free and overall survival. Recruitment started in August 2018 and is ongoing.

## Introduction

Up to 60% of patients diagnosed with head and neck squamous cell carcinoma (HNSCC) present with locally advanced disease (1). In this patient group, multidisciplinary therapy is generally recommended with either surgery followed by adjuvant radiotherapy (RT) or radiochemotherapy (aRCT) or definitive RCT (1). Patients with locally advanced head and neck squamous cell carcinoma often experience relapse, the cause of relatively worse survival. Microscopically involved resection margins (R1) and extracapsular spread (ECS) of lymph node metastases are the most significant prognostic factors (“high risk”) for poor outcome. Close margins (R0 <5 mm) and the presence of ≥ 2 lymph node metastases represent “intermediate-risk” features predictive for worse disease control (2, 3). Unfortunately, and despite advantages in imaging diagnostics and therapy regimens in the curative setting, even completely resected high and intermediate risk HNSCC are prone to loco-regional recurrence and/or distant metastasis and about 45 to 50% of patients experience such an event or even death within 24 months (1–4). The surgical concepts for advanced resectable HNSCC have not changed significantly within the last 20 years. In experienced centers, additional techniques like transoral laser or robotic microsurgery (TLM, TORS) to conventional surgical approaches and neck dissection are standard procedures for treatment of advanced resectable HNSCC. The addition of concomitant cisplatin to aRT as aRCT improves outcome in patients with intermediate and high risk who are fit to receive chemotherapy (4). The HPV-related prognostic impact in oropharyngeal cancer did not change the standard treatment until yet and is currently under investigation in clinical trials. Despite the currently available treatment options for patients with locally advanced HNSCC, there is a need for better therapies in the curative setting for this patient population. Strategies to improve survival with intensified therapy for patients with high-risk HNSCC have been limited by excessive toxicity (5).

Since the first publication of the beneficial use of checkpoint inhibitors in HNSCC, these have been explored in different clinical settings. Pembrolizumab is a potent and highly selective humanized, monoclonal anti–programmed death receptor-1 (PD-1) antibody that has demonstrated robust antitumor activity and an acceptable safety profile in different cancer entities (6). Pembrolizumab is approved for recurrent/metastatic HNSCC with disease progression on or after platinum-containing chemotherapy and previously untreated recurrent/metastatic HNSCC. The approval is based on the positive results of the KEYNOTE-040 trial, evaluating pembrolizumab monotherapy in patients whose HNSCC progressed after platinum therapy (7), and the KEYNOTE-048 trial, evaluating pembrolizumab, alone or in combination with chemotherapy, in patients who did not previously receive systemic therapy for recurrent/metastatic disease (8). Although pembrolizumab is approved for recurrent/metastatic disease, its role in the primary management of locally advanced HNSCC is not defined. The relevant prolongation of overall survival (OS) and favorable safety profile of pembrolizumab in patients with recurrent or metastatic HNSCC (7, 8) support the further evaluation of pembrolizumab in earlier stages of disease. The ADRISK trial wants to investigate if the addition of pembrolizumab to aRCT with cisplatin improves event-free survival (EFS) compared with aRCT alone in locally advanced intermediate- and high-risk HNSCC.

## Methods

### Study design

**Figure 1:**
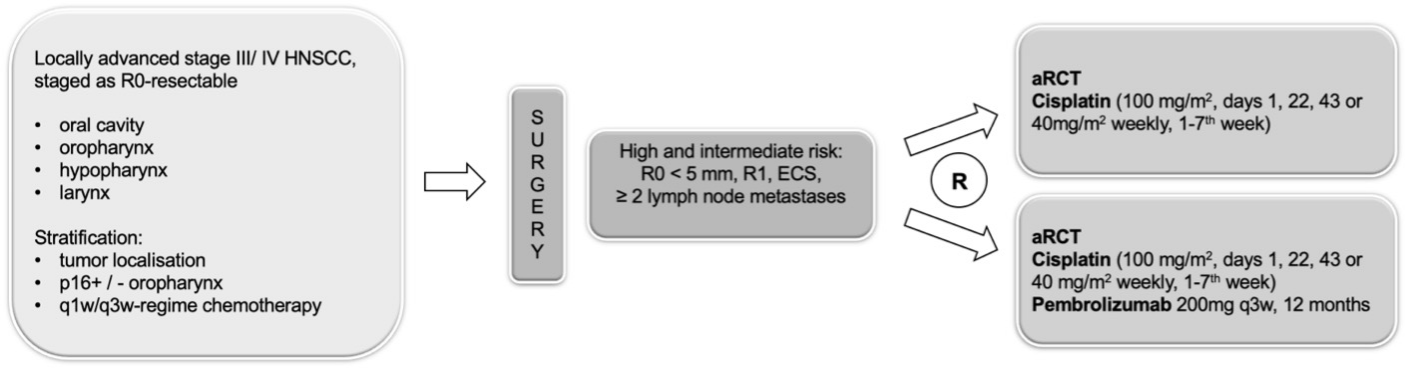
Study design of the ADRISK trial.

ADRISK is a prospective, randomized, open-label, two-armed phase II multicenter superiority trial within the German Interdisciplinary Study Group of German Cancer Society (IAG-KHT). Patients with primary resectable stage III and IV (according to 7^th^ edition of TNM classification) HNSCC of the oral cavity, oropharynx, hypopharynx and larynx with pathologic high (R1, extracapsular nodal extension) or intermediate risk (R0 <5 mm; N≥2) after surgery who are eligible for cisplatin-based aRCT after surgery will receive either standard aRCT with cisplatin versus the same treatment + pembrolizumab (200 mg iv, in 3-week cycle, max. 12 months) (**Figure 1**). This clinical trial has been approved by the ethics committee of the University of Leipzig (076/18-ff).

All persons participating in the conduct of the trial (sponsor, authorized representative of the sponsor, investigators, etc.) commit themselves to observe the Declaration of Helsinki of the WMA in its current version, as well as all pertinent national laws and the ICH guideline for Good Clinical Practice (GCP) ICH E6(R2) (EMA/CHMP/ICH/135/1995) issued in June 2017. The trial is registered on the clinicaltrials.gov database (NCT03480672).

### Study objectives

The primary objective of ADRISK is to show that addition of pembrolizumab to aRCT with cisplatin improves EFS compared with aRCT alone in locally advanced intermediate and high-risk HNSCC after surgery. The primary endpoint EFS is defined as time from randomization to the first event, i.e.:

- locoregional or distant recurrence (relapse),
- occurrence of further malignant disease (independent from localization and type),
- death from any cause, and
- initiation of a new anti-cancer treatment without a previous event (primarily in case of safety concerns/toxicities observed which must be addressed in the patient**’**s treatment).

Secondary goals of the trial are to show that addition of pembrolizumab to aRCT with cisplatin improves OS compared with aRCT alone in locally advanced intermediate and high-risk HNSCC and to assess toxicity and show that addition of pembrolizumab to aRCT with cisplatin is safe in terms of toxicity (early *and* late toxicity). The duration of patients**’** observation is planned for at least 24 months post randomization, but preferably until LPLV (last patient**’**s last visit after 24 months of observation) to collect endpoint-events as comprehensive as possible.

Within the trial PD-L1 and p16 (only at tumor site oropharynx) expression in tumor tissue will be characterized by immunohistochemistry to explore the relationship between tumor PD-L1 and p16 expression and response to treatment with pembrolizumab. Therefore, material collected during the tumor resection will be analyzed locally at trial site and validated by reference pathologists in the central lab.

### Trial population

A sample of 240 patients was planned to be randomized at a 1:1 ratio (about half of the patients to each treatment arm) after stratification for q1w/q3w aRCT regimen and localization of tumor as well as p16-status in oropharynx cancer.

#### Inclusion criteria

1. Macroscopically complete resection of newly diagnosed (not recurrent, not secondary primary) advanced squamous-cell carcinoma arising in the oral cavity, oropharynx, larynx, or hypopharynx
2. Advanced stage III, IVA/B HNSCC according to the TNM classification version 7th edition
3. Eastern Cooperative Oncology Group (ECOG) performance status of 0 to 1; performance status allows adjuvant chemo radiation with cisplatin.
4. Had either intermediate or high-risk characteristics, i.e. any or all of the following:
 - histologic evidence of invasion of two or more regional lymph nodes
 - extracapsular extension of nodal disease,
 - microscopically involved mucosal margins of resection (R1) or margins of resection < 5mm (R0)
5. Had pathological histologic assessment of p16 (only oropharyngeal carcinoma)
6. Be > 18 years of age
7. Written informed consent
8. Demonstrate adequate organ function as defined in **Table 1**; all baseline labs should be performed within 10 days of treatment initiation.
9. Female subject of childbearing potential should have a negative pregnancy test within 3 days prior to receiving the first dose of study medication.
10. Female subjects of childbearing potential must be willing to use an adequate method of contraception, for the course of the study through 120 days after the last dose of study medication.
11. Reproductive male subjects must agree to use an adequate method of contraception, starting with the first dose of study therapy through 120 days after the last dose of study therapy.

**Table 1:** Adequate organ function laboratory values.

System	Laboratory value
**Hematological** Absolute neutrophil count (ANC) Platelets Hemoglobin	≥1,500/μL 1≥ 100,000 μL ≥9 g/dL or ≥5.6 mmol/L without transfusion or EPO dependency (Criteria must be met within last 2 weeks)
**Renal** Serum creatinine **OR** Measured or calculated creatinine clearance (GFR can also be used in place of creatinine or CrCl)	≤1.5x upper limit of normal (ULN) **OR** ≥60 mL/min for subject with creatinine levels > 1.5 X institutional ULN
**Hepatic** Serum total bilirubin AST (SGOT) and ALT (SGPT) Albumin	≤ 1.5x ULN **OR** Direct bilirubin ≤ ULN for subjects with total bilirubin levels > 1.5 ULN ≤ 2.5x ULN **OR** **≤** 5x ULN for subjects with liver metastases >2.5 mg/dL
**Coagulation** International Normalized Ratio (INR) Activated Partial Thromboplastin Time (aPTT)	≤1.5x ULN unless subject is receiving anticoagulant therapy as long as aPTT is within therapeutic range of intended use of anticoagulants

#### Exclusion criteria

The subject must be excluded from participating in the trial if the subject meets any of the following criteria:

1. Concurrent participation in any other interventional clinical trial or participation in any other interventional trial within one month before enrolment into this trial.
2. Diagnosis of immunodeficiency or is receiving systemic steroid therapy or any other form of immunosuppressive therapy within 7 days before enrolment into this trial.
3. Known history of active TB (Bacillus tuberculosis)
4. Hypersensitivity to pembrolizumab or comparable medicinal products or any of its excipients.
5. Prior anti-cancer monoclonal antibody (mAb) therapy within one month before enrolment into this trial or who has not recovered (i.e., ≤ Grade 1 (NCI CTCAE Grade) at baseline) from adverse events due to agents administered more than one month earlier.
6. Prior chemotherapy, targeted small molecule therapy, or radiation therapy within one month before enrolment into this trial or who has not recovered (i.e., ≤ grade 1 (NCI CTCAE Grade) at baseline) from adverse events due to a previously administered agent.
  a.) Subjects with ≤ Grade 2 (NCI CTCAE Grade) neuropathy are an exception to this criterion and may qualify for the study.
  b.) If subject received major surgery, they must have recovered adequately from the toxicity and/or complications from the intervention prior to starting therapy.
7. Known additional malignancy that is progressing or requires active treatment. Exceptions include basal cell carcinoma of the skin or squamous cell carcinoma of the skin that has undergone potentially curative therapy or *in situ* cervical cancer.
8. Active autoimmune disease that has required systemic treatment in the past 2 years prior to enrolment (i.e. with use of disease modifying agents, corticosteroids or immunosuppressive drugs). Replacement therapy (e g., thyroxine, insulin, or physiologic corticosteroid replacement therapy for adrenal or pituitary insufficiency, etc.) is not considered a form of systemic treatment.
9. Evidence of interstitial lung disease or history of (non-infectious) pneumonitis that required steroids within the last 6 months before enrolment into this trial, or current pneumonitis.
10. Active infection requiring systemic therapy.
11. Suspected lack of compliance
12. Is pregnant or breastfeeding, or expecting to conceive or father children within the projected duration of the trial, starting with the baseline visit through 120 days after the last dose of trial treatment.
13. HIV, HBV or HCV infection
14. Application of a live vaccine within one month of enrolment.
15. Hypersensitivity to cisplatin or any of its excipients
16. Any potential relationship to the investigator/his deputy or to medical staff of the study team, to the coordinating investigator or is an employee of the study site

### Interventions

#### Experimental intervention

Patients will receive pembrolizumab intravenously for 12 months, in 3-week cycle (q3w) 200 mg, in combination with standard treatment (adjuvant radio-chemotherapy).

Radiotherapy: standard adjuvant radiotherapy (e.g. pN0 50 Gy; pN1 56 Gy; pECS + primary 66 Gy)

Chemotherapy with cisplatin: (according to the respective standard of the center)

- Cisplatin cumulative dose 300 mg/m^2^ body surface according to Cooper et al./Bernier et al. (2, 3) or
- Cisplatin cumulative dose: 280 mg/m^2^ body surface, e.g. Cisplatin 40 mg/m^2^ iv, weekly in 1-7^th^ week of treatment

#### Control intervention

Standard treatment (adjuvant radio-chemotherapy)

### Trial treatments

#### Radiochemotherapy

The standard treatment has to be started at day 1 (week 1) in both treatment arms and should follow national guidelines. All patients will be treated as respective clinic standard and receive best standard care.

#### Radiotherapy

Radiation is delivered using IMRT in respect to the clinic standard. Planning and delivery of IMRT can be performed using sequential boost (SeqB) or simultaneous integrated boost (SIB). Following is an example how the process could be using SeqB: All patients will be immobilized in supine position in thermoplastic head, neck, and shoulder masks in order to conduct treatment-planning CT scans (slice thickness 3 mm) to define the target volumes and the organs at risk (OAR). Radiotherapy will be given at linear accelerators or helical tomotherapy by use of isocentric techniques with intensity modulated radiotherapy (IMRT) with 6 MV photon beams (9). All patients receive conventional daily single doses of 2 Gy, and five fractions per week. The target volumes and doses are determined from surgical results, and from CT/MRI. Three clinical target volumes (CTV) are defined: CTV 66 (total dose 66 Gy) for high-risk regions (R1, ECS), CTV 56 (total dose 56 Gy) for intermediate-risk regions (tumor bed (R0), involved nodes without ECS), and CTV 50 (total dose 50 Gy) for low-risk regions (i.e., pN0 or nodes down to the clavicles in both groups). Planning target volume (PTV) was defined as the CTV plus a 5- to 10-mm margin to compensate variables of treatment setup and motion of internal organs (10, 11). In the case of anatomical boundaries and to the spinal cord, the distances are to keep personalization. PTV should not go outside the skin surface, and can be retraced from surface by 3 mm. In general, the volume definition of cervical lymph node levels should be performed using the RTOG head and neck lymph node atlas (www.rtog.org) and additional recommendations for delineation and selection of elective neck levels (12). The OAR contouring should be done according to the consensus guidelines of DAHANCA, EORTC, GORTEC, HKNPCSG, NCIC CTG, NCRI, NRG Oncology and TROG (13).

#### Chemotherapy

The q1w and q3w schedule will be allowed in the ADRISK and may be applied according to the respective standard of the center. The dosing and timing of chemotherapy to be used in this trial is outlined in **Table 2**. These options were chosen since the q3w schedule is not very common in Germany. However, the balancing of the cisplatin/RT doses will be reviewed permanently to rule out bias between the arms and the cisplatin schedule is used as a stratification criterion. A minimum of 200 mg/m^2^ cisplatin should be aimed at in all patients. An intravenous infusion (volume replacement) will be given as supportive therapy. All patients receive adequate antiemetic prophylaxis. The choice between q1w and q3w is left to the appraisal of the radiation oncologist, considering age, weight loss before radiation, creatinine clearance and performance status. In practice, various radiotherapists systematically use q1w cisplatin, and others use q3w cisplatin. In case of poor radiochemotherapy tolerability, the patients have to be hospitalized or receive a nasogastric intubation.

**Table 2:** Cisplatin treatment.

Drug	Dose/Body Surface Area	Frequency Dose/Body Surface Area	Route of Administration	Regimen/Treatment Period
Cisplatin	300 mg/m^2^	q3w 100 mg/m^2^	inravenous infusion	days 1, 22, and 43
Cisplatin	280 mg/m^2^	q1w 40 mg/m^2^	intravenous infusion	days 1, 8, 15, 22, 29, 36, 43 1-7th week

Patients must meet the following criteria before each new cycle:

• No hematotoxicity of grade ≥2 (i.e. neutrophils >1500/μL, platelets >100.000/μL, hemoglobin ≥9 g/dl, if required after transfusion)
• Recovery from any treatment-related grade 3/4 non-hematological toxicity (except alopecia) to baseline or ≤grade 1
• No ongoing requirement for anti-diarrheic treatment
• Bilirubin ≤3.0 x ULN
• Transaminases ≤2.5 x ULN
• No peripheral neurotoxicity of grade 3
• Creatinine clearance ≥60 mL/min
• No treatment delay of more than 3 weeks

Patients not meeting the above criteria on the date scheduled for the new cycle must suspend treatment with the anticancer drugs until they meet the above criteria. In the case of a cisplatin intolerance during RCT, it may be possible to switch to carboplatin. The administration of mitomycin is not permitted.

#### Pembrolizumab treatment

The experimental treatment to be used in this trial is outlined in **Table 3**. First cycle of pembrolizumab starts on day 1 of aRCT. Pembrolizumab should be administered at least 30 minutes prior to premedication for the chemotherapies. Furthermore, pembrolizumab should be administered prior to chemotherapy. Adverse events (both non-serious and serious) associated with pembrolizumab exposure may represent an immunologic etiology. These adverse events may occur shortly after the first dose or several months after the last dose of treatment. Pembrolizumab must be withheld for drug-related toxicities and severe or life-threatening AEs. Dosing interruptions are permitted in the case of medical/surgical events or logistical reasons not related to study therapy (e.g., elective surgery, unrelated medical events, patient vacation, and/or holidays). Subjects should be placed back on study therapy within 3 weeks of the scheduled interruption, unless otherwise discussed with the sponsor. The reason for interruption should be documented in the patient’s study record.

**Table 3:** Pembrolizumab treatment.

Drug	Dose	Frequency Dose	Route of Administration	Regimen/Treatment Period
Pembrolizumab	200mg	q3w	intravenous infusion	Day 1 of each 3 week cycle over 51 weeks

Pembrolizumab should be administered on day 1 of every three-week cycle, repeat every 3 weeks (+/- 3 days; due to administrative reasons) until regular end of treatment (18 cycles, week 52), disease recurrence or premature termination for other reasons. All trial treatments can be administered on an outpatient basis, depending on the respective standard of the center. (Potential) outpatient basis means that trial treatments always take place in a clinic with medical personnel that is sufficiently trained for such emergency situations (e.g. severe infusion reactions). The appropriate emergency care is guaranteed and will be provided immediately. Pembrolizumab 200 mg will be administered as 30 minutes intravenous infusion every 3 weeks. Sites should make every effort to target infusion timing to be as close to 30 minutes as possible. However, given the variability of infusion pumps from site to site, a window of -5 minutes and +10 minutes is permitted (i.e., infusion time is 30 minutes: -5 min/+10 min).

### Informed consent procedure

Before enrolment in the ADRISK trial, the patient will be educated that participation is voluntary and that he/she may withdraw from the clinical trial at any time without having to give reasons and without penalty or loss of benefits. The patient will be informed by the treating physician about the treatment methods to be compared, the expected risks and benefits and alternative treatment methods. The patient should be provided enough time to think about the participation in the clinical trial. Before any trial specific actions, the patient’s written consent must be obtained by dating and signing the informed consent form by the trial patient and the physician.

### Randomization process

Randomization of patients between experimental and control arm is performed centrally *via* a secure web-based tool using a modified minimization procedure with stochastic component according to Pocock (14) in a 1:1 proportion. Stratification is performed using the tumor localizations: 1) oral cavity, 2) oropharynx, 3) larynx, or 4) hypopharynx. For patient with oropharyngeal carcinoma, the p16-marker is generally determined in routine procedures and will be used as sub-classifier 2+) and 2-) so that 5 strata will be used in total. Furthermore, a stratification according to the q3w vs. q1w schedule of chemotherapy will be applied. Randomization will be balanced according to trial site as well. The result of the successful randomization is immediately available *via* the randomization tool and is forwarded automatically to the investigator and the Clinical Trial Center Leipzig.

### Schedule of trial procedures

**Table 4** summarizes the trial procedures to be performed as planned or at unscheduled time points if deemed clinically necessary by the investigator.

**Table 4:** Schedule of trial procedures.

Trial period	Baseline (BL)	V1 – V4 Safety Follow-up	C1 – C18 Pembrolizumab treatment		V5 – V12 (V13 - V16 optional) Efficacy/Safety Follow-up	SV1, SV2… Survival Follow-up
Time	ALL patients		only patients in EXPERIMENTAL arm		ALL patients	ALL patients AFTER disease RECURRENCE
day **–** 10 - day 0	weeks 1, 4, 7, 10 (+/- 3 days)	**day 1** (week 1) **of aRCT** Cycle 1	week 4 - 52 (every 3 weeks) (+/- 3 days) Cycles 2 - 18	month 3, 6, 9, 12 (+/- 7 days) month 15, 18, 21, 24 (+/- 14 days) every 6 months (+/- 28 days) thereafter - optional	every 6 months after recurrence
Administrative procedures						
Informed consent	X					
Eligibility criteria	X		X^1^			
Demographics/Medical history	X					
Registration Randomization	X^2^					
Prior/concomitant medication	X	X^3^	X	X	X	
Treatment						
Adjuvant radio-chemotherapy		X				
Pembrolizumab			X	X		
Anticancer therapy after recurrence						X
Clinical Procedures/Assessments						
Adverse Events		X^3^	X	X	X^4^	
Physical examination	X	X^3^	X	X	X	
Vital signs	X	X^3^	X	X	X	
ECOG status	X	X^3^	X	X	X	
Laboratory measurements	X	X^3^	X	X	X^5^	
Survival status; locoregional or distant recurrence, initiation of a new anti-cancer treatment		X^3^	X	X	X	X
Efficacy measurements						
Clinical endoscopic assessment	X				X	
CT or MRI Head and Neck	X				X^6^	
CT chest	X				X^7^	

V= visit; C= 3-week cycle (q3w) pembrolizumab infusion (and aRCT) starting on day 1 of each cycle; SV, survival visit.

^1^If results of laboratory procedures done at Baseline Visit are older than 10 days, new blood sampling are necessary prior to start of treatment with pembrolizumab. Furthermore, pregnancy test must be negative within 3 days prior to receiving the first dose of study medication.

^2^Randomization must be done by the day 1 (start of the trial treatment) at the latest.

^3^In control arm all clinical procedures/assessments should correspond with the experimental arm (three-weekly +/- 3 days) up to and including first efficacy follow-up (month 3 after randomization).

^4^In experimental arm: until 90 days following cessation of treatment, or up to initiation of a new anti-cancer treatment; in control arm: until 1^st^ efficacy-FU (month 3).

^5^Only at safety follow-up at week 56 (+/- 7 days) or at premature treatment ermination (one month (+/- 7 days) after termination) - in the experimental arm.

^6^Every 6 months only.

^7^Every 12 months only.

### Plan for further treatment

Radiochemotherapy treatment in both groups takes maximal seven weeks. Pembrolizumab will be given in the experimental group over a period of 51 weeks. If patient responds to treatment no further therapy is necessary. If a patient experiences a disease recurrence (primary endpoint) detected by radiographic imaging, standard of care treatment outside the trial protocol is necessary, which is up to the discretion of the treating physician/investigator. Further observation for OS takes place.

### Statistical methods

#### Estimation of sample size

We expected a 2yr EFS of 55% with aRCT alone based on Cooper et al. (3, 15) and Bernier et al. (2) and assumed proportional hazards. A hazard ratio of 0.6 corresponds to an increase in EFS rates (at ~2 years) from e.g. 55 to 70% - an effect size worth detecting. Requiring a 5% one-sided significance level and 80% power, we need to observe at least 93 events for inference with the log rank test or by Cox regression. Since the effect of the experimental treatment is expected to become apparent only after 3-4 months, and the power may be slightly reduced, we set a target of 100 events. The number of patients needed to observe this count depends on the times of accrual, follow-up as well as the strategy of FUP. After modeling the expected information flow and calculating the expected observed events since start of accrual (using the pooled EFS curve in Cooper et al. (3)), we specified the end of study after 2 years of last patient’s observation or when at least 100 events have been observed before that time.

#### Analysis population

The full analysis set (FAS, also called intention-to-treat (ITT) population) includes all randomized patients who start with the trial-related interventions. Randomized patients who withdraw their informed consent before the 1st trial intervention takes place will be excluded from the FAS.

The per-protocol (PPS) set is defined by all patients belonging to the ITT without major violations of the study protocol. Beside violations of eligibility criteria and patients not treated as randomized further protocol violations occurring during study conduct are reviewed and classified as major in the statistical analysis plan independently from arm allocation.

The safety population is defined by all randomized patients belonging to the FAS. In safety analyses, patients will be classified whether or not they received at least a single Pembrolizumab dose, irrespective of the randomized group allocation.

#### Planned methods for analysis

Confirmatory analysis follows the intention to treat principle and will be based on the full analysis set. Time to event endpoints will be analyzed by Cox regression with arm as fixed effects, stratification criteria as fixed and center as random effect. The occurrence of safety endpoints (AE by Preferred Term/occurrences of toxicities of grades ≥3) will be compared by chi-square tests. Odds ratios with 95% confidence intervals will be provided. Serious adverse events will be reported descriptively (including a clinical causality assessment) by System Organ Class and Preferred Term according to MedDRA. In case of relevant frequencies of SAE and classified serious adverse reactions (by Preferred Term) exact Fisher test will be applied on SAE/SAR occurrences. No confirmatory subgroup analyses are planned. PD-L1 status will be analyzed in exploratory analyses. Safety issues will be carefully monitored. Regular safety analyses will be performed and presented to the Data-Monitoring Committee (DMC). Statistical monitoring will focus on data quality assurance. All analyses will be pre-specified in a detailed statistical analysis plan, which will be finalized before data base closure. The trial will be reported according to CONSORT criteria.

#### Final analysis

Final analysis will be performed when the data of all enrolled patients have been collected, all queries have been the resolved and the data base has been closed. No formal interim analysis on efficacy is planned.

## Discussion

For locoregional advanced, but resectable HNSCC, the current standard of care includes definitive surgical resection and risk-adapted aRT or aRCT, or definite chemoradiation. The choice of treatment is subject to regional preferences, in Germany initial treatment is traditionally dominated by surgery (16). In the last years, immunotherapy became an effective fourth treatment modality in patients with HNSCC. Based on the results from the KEYNOTE-012, -040 and CheckMate-141 trials, nivolumab and pembrolizumab received first approval in HNSCC patients as monotherapy for the treatment of recurrent or metastatic HNSCC progressing on or after platinum-containing chemotherapy from the FDA and EMA (8, 17, 18). In 2019, results from the KEYNOTE-048 trial showed that pembrolizumab monotherapy improved OS versus the current standard of care, the EXTREME regimen (carboplatin/cisplatin, 5-FU, cetuximab + cetuximab maintenance), in HNSCC patients with combined positive score (CPS) of 1 or more and was non-inferior in the total population (7). Pembrolizumab with chemotherapy was superior in the total population of the study regarding OS compared to the EXTREME regimen (7). This led to an extension of the approval of pembrolizumab mono- and combination therapy as first-line treatment without prior platinum-based chemotherapy in patients with CPS≥1.

After demonstrating the efficacy and safety of immune checkpoint inhibitors in recurrent/metastatic HNSCC (7, 8, 17, 18), investigators have begun to assess whether immunotherapy might play a role as part of curative-intent treatment regimens. Unfortunately, two phase III trials investigating the addition of checkpoint inhibitors to concurrent chemoradiation were negative. The Javelin 100 phase III trial randomizing patients with locoregionally advanced HNSCC to cisplatin-radiotherapy with avelumab or placebo did not achieve the primary objective of prolonging progression-free survival with avelumab plus chemoradiation followed by avelumab maintenance (19). The KEYNOTE-412 trial evaluating pembrolizumab with concurrent RCT followed by pembrolizumab as maintenance therapy did also not meet its primary endpoint of EFS for the treatment of patients with unresected locally advanced HNSCC. The experimental arm was associated with a favorable trend toward improved EFS versus RCT and placebo, but the difference did not reach statistical significance (20). However, until now, there were no results of randomized trials investigating the addition of checkpoint inhibitors to adjuvant chemoradiation and to our knowledge, ADRISK is the only multicenter, randomized trial analyzing the addition of pembrolizumab to aRCT in patients with locally advanced HNSCC without prior neoadjuvant treatment. Actually, the field of HNSCC treatment is moving fast and it is expected that initial treatment will be complemented by immunotherapies, at least in defined subsets of patients.

## Conclusion

ADRISK is a randomized, phase II trial aiming to investigate if the addition of pembrolizumab to aRCT with cisplatin improves EFS and OS and is safe in terms of toxicity compared with aRCT alone in locally advanced intermediate and high-risk HNSCC. The ADRISK trial started recruitment in August 2018 and plans to include 240 patients.

## Data availability statement

The original contributions presented in the study are included in the article/supplementary material. Further inquiries can be directed to the corresponding author.

## Ethics statement

The studies involving human participants were reviewed and approved by ethics committee of the University of Leipzig (076/18-ff.; date of approval: 2018-06-21). The patients/participants provided their written informed consent to participate in this study.

## Author contributions

Conceptualization, SW, GW, AS and AD. Methodology, SW, GW, JV, AF, AS and AD. Writing – original draft, SW, GW and AD. Writing – review & editing, JV, KV, AF, TK, FL, AS, PH, PB, FB, HK, GM, MP, MM, VL, NR, CS, MB, SD, TG, GH, OG-L, US, MG, MH, FS, BT and AS. All authors contributed to the article and approved the submitted version.
